# The impact of music experience on hippocampal subfields and cognitive function in older adults at risk for dementia

**DOI:** 10.1002/alz.093306

**Published:** 2025-01-09

**Authors:** Nicole Espinosa, Hannes Almgren, Andrew C McKinnon, Sharon L Naismith

**Affiliations:** ^1^ University of Sydney, Sydney, NSW Australia; ^2^ The University of Sydney, Sydney, NSW Australia; ^3^ Race Against Dementia ‐ Dementia Australia Research Initiative, Sydney, NSW Australia; ^4^ Healthy Brain Ageing Program, Brain and Mind Centre, University of Sydney, Sydney, NSW Australia; ^5^ Charles Perkins Centre, University of Sydney, Sydney Australia

## Abstract

**Background:**

It has been argued that engaging in musical activities throughout one's lifespan can promote neuroplasticity and protect against age‐related cognitive decline. Most of the research on this topic has been performed on professional musicians; however, general music experience across the lifespan may also have a protective effect on cognitive performance and brain health in non‐professional musicians. This study investigates the effects of lifetime music experience on hippocampal subfield grey matter (GM) volumes and memory and executive functioning in older adults at risk of dementia.

**Method:**

168 individuals aged ≥50 years (mean age = 68.83, females = 67.9%, MMSE = 28.99 ) with (n = 87) and without (n = 81) a history of music experience and with a MMSE ≥24 were recruited from a memory clinic. Participants underwent tests of memory (Logical Memory subtest of the Wechsler Memory Scale‐III and the Rey Auditory Verbal Learning Test; RAVLT) and executive functioning (the DKEFS Colour Word Interference Test condition 3; CWIT, and the Trail Making Test part B; TMT), as well as MRI data acquisition of the hippocampus (T1 and high‐res hippocampus T2) from which the CA1, dentate gyrus (DG) and subiculum were computed (and corrected for intracranial volume) using the automated segmentation of hippocampal subfields (ASHS) software. Multiple Pearson’s partial correlational analyses adjusting for age were carried out to examine the association between music experience, hippocampal subfields, and cognition. All statistical tests were two‐tailed (alpha = 0.05).

**Result:**

Pearson's partial correlation coefficient indicated that music experience was associated with higher scores in both tests of executive functioning, the TMT (r = 0.172; p = 0.028) and CWIT (r = 0.196; p = 0.012). However, memory was not significantly correlated with music experience. There were no differences in hippocampal subfield volumes between those who did and did not have musical experience.

**Conclusion:**

Older adults with music experience showed significantly better executive functioning compared to those without. However, this improvement was not associated with any differences in hippocampal subfield volumes. Further research is needed to fully understand the effect of music experience on other MRI markers.

